# Urban and Transport Planning Related Exposures and Mortality: A Health Impact Assessment for Cities

**DOI:** 10.1289/EHP220

**Published:** 2016-06-27

**Authors:** Natalie Mueller, David Rojas-Rueda, Xavier Basagaña, Marta Cirach, Tom Cole-Hunter, Payam Dadvand, David Donaire-Gonzalez, Maria Foraster, Mireia Gascon, David Martinez, Cathryn Tonne, Margarita Triguero-Mas, Antònia Valentín, Mark Nieuwenhuijsen

**Affiliations:** 1 ISGlobal, Centre for Research in Environmental Epidemiology (CREAL), Barcelona, Spain; 2 Universitat Pompeu Fabra (UPF), Barcelona, Spain; 3 CIBER Epidemiología y Salud Pública (CIBERESP), Madrid, Spain; 4 Physical Activity and Sports Sciences Department, Fundació Blanquerna, Barcelona, Spain; 5 Swiss Tropical and Public Health Institute, Basel, Switzerland; 6 University of Basel, Basel, Switzerland

## Abstract

**Background:**

By 2050, nearly 70% of the global population is projected to live in urban areas. Because the environments we inhabit affect our health, urban and transport designs that promote healthy living are needed.

**Objective:**

We estimated the number of premature deaths preventable under compliance with international exposure recommendations for physical activity (PA), air pollution, noise, heat, and access to green spaces.

**Methods:**

We developed and applied the Urban and TranspOrt Planning Health Impact Assessment (UTOPHIA) tool to Barcelona, Spain. Exposure estimates and mortality data were available for 1,357,361 residents. We compared recommended with current exposure levels. We quantified the associations between exposures and mortality and calculated population attributable fractions to estimate the number of premature deaths preventable. We also modeled life-expectancy and economic impacts.

**Results:**

We estimated that annually, nearly 20% of mortality could be prevented if international recommendations for performance of PA; exposure to air pollution, noise, and heat; and access to green space were followed. Estimations showed that the greatest portion of preventable deaths was attributable to increases in PA, followed by reductions of exposure to air pollution, traffic noise, and heat. Access to green spaces had smaller effects on mortality. Compliance was estimated to increase the average life expectancy by 360 (95% CI: 219, 493) days and result in economic savings of 9.3 (95% CI: 4.9, 13.2) billion EUR/year.

**Conclusions:**

PA factors and environmental exposures can be modified by changes in urban and transport planning. We emphasize the need for a) the reduction of motorized traffic through the promotion of active and public transport and b) the provision of green infrastructure, both of which are suggested to provide opportunities for PA and for mitigation of air pollution, noise, and heat.

**Citation:**

Mueller N, Rojas-Rueda D, Basagaña X, Cirach M, Cole-Hunter T, Dadvand P, Donaire-Gonzalez D, Foraster M, Gascon M, Martinez D, Tonne C, Triguero-Mas M, Valentín A, Nieuwenhuijsen M. 2017. Urban and transport planning related exposures and mortality: a health impact assessment for cities. Environ Health Perspect 125:89–96; http://dx.doi.org/10.1289/EHP220

## Introduction

By 2050, nearly 70% of the global population is projected to live in urban environments (United Nations 2014). Cities can be beneficial for people’s well-being because they provide innovation, access to goods and services, and they facilitate social interaction (United Nations 2014). Some aspects of urban life, however, such as a sedentary lifestyle; increased exposure to air pollution, noise, and heat; and a lack of green space, can have detrimental effects on health and can increase premature mortality [Gascon et al. 2016; Guo et al. 2014; Halonen et al. 2015; Woodcock et al. 2011; World Health Organization (WHO) Regional Office for Europe 2014b].

Physical inactivity and ambient air pollution are estimated to cause more than five million premature deaths each year worldwide and rank among the leading risk factors in the Global Burden of Disease Study (GBD 2013 Risk Factors Collaborators et al. 2015). Car-centric city designs typical of preceding decades have little space assigned for green infrastructure, despite the increasingly known benefits for physical and mental health (Gascon et al. 2016).

In addition to being the main source of air pollution in urban areas, motorized road traffic exposes an estimated 40% of Europeans to daytime noise levels exceeding the WHO recommended threshold of 55 dB (WHO 1999); it also produces anthropogenic heat that together with the reradiation effects of dense urban structures can amplify urban summer temperatures, resulting in urban heat islands (Zhao et al. 2014). Reducing exposure to urban environmental hazards, increasing exposure to green spaces, and promoting physical activity (PA) may be achievable through community-level interventions such as health-promoting urban and transport planning.

We aimed to estimate the mortality burden associated with exposures related to current urban and transport planning. For this purpose, we developed the Urban and TranspOrt Planning Health Impact Assessment (UTOPHIA) model and conducted a health impact assessment (HIA) for Barcelona, Spain. We estimated the impact of meeting the international recommendations for performance of PA; exposure to air pollution, noise and heat; and access to green spaces on preventable natural all-cause mortality, life expectancy, and economic savings.

## Methods

### Study Setting

As of 2012, Barcelona, which is located on the northeastern coast of Spain, had 1,620,943 inhabitants living in an area of 101 km^2^ (Barcelona City Council 2012). Barcelona has a Mediterranean climate with an annual mean temperature of 18°C through mild winters and hot, humid summers (Barcelona City Council 2012). Temperatures in the densely inhabited center of Barcelona can be ≤ 8°C higher compared with the spacious surrounding areas because of the urban heat island effect (Moreno-Garcia 1994). Air pollution and noise levels are among the highest in Europe owing to Barcelona’s high population and traffic density, large share of diesel-powered vehicles, low precipitation, and an urban design of narrow street canyons framed by semi-tall buildings of 5–6 stories (Nieuwenhuijsen et al. 2014). In turn, green space is mainly located at the hilly west side of Barcelona, and only 6.8 m^2^ of green space is available per resident on a city-wide average (Barcelona City Council 2012).

### HIA Methodology: UTOPHIA

We conducted an HIA analysis at the Barcelona census-tract level (*n* = 1,061) using data from 2012. The analysis estimated the impact on natural all-cause mortality for Barcelona residents ≥ 20 years of age (*n* = 1,357,361) under compliance with international exposure level recommendations. The 2012 natural all-cause mortality rate for Barcelona residents ≥ 20 years of age was 1,108 deaths/100,000 persons after excluding external causes of death (see Tables S1 and S2) (Agència de Salut Pública de Barcelona 2012).

**Figure 1:**
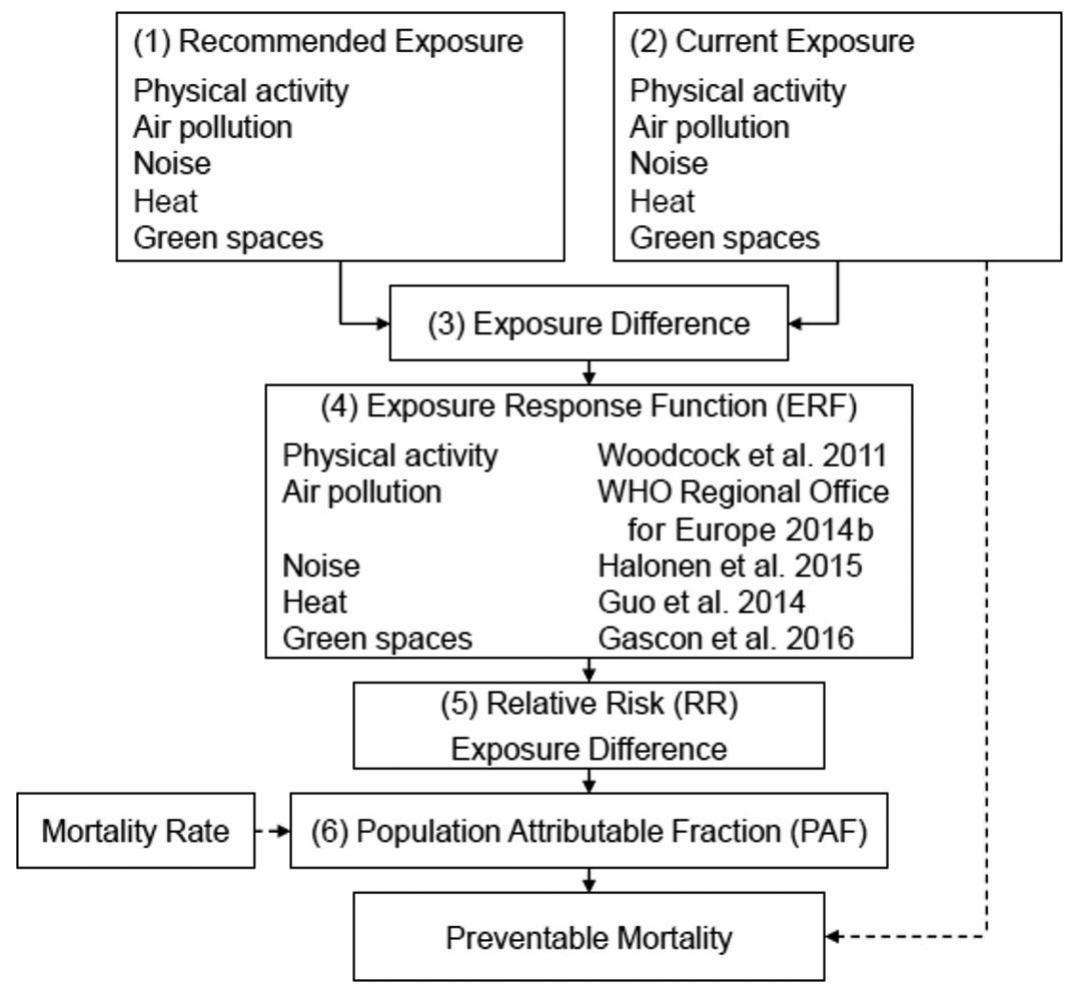
Conceptual framework of the Urban and TranspOrt Planning Health Impact Assessment (UTOPHIA) tool. (*1*) Recommended exposure level; (*2*) current exposure level; (*3*) exposure difference between recommended and current exposure level; (*4*) exposure response function (ERF) quantifying association between exposure and mortality; (*5*) relative risk (RR) corresponding to exposure difference; (*6*) population attributable fraction (PAF) corresponding to exposure difference.

We developed the UTOPHIA tool following quantitative HIA methodology (Figure 1) [WHO and United Nations Environment Programme (UNEP) 2015] as follows: *a*) We obtained recommended exposure levels (“counterfactual exposure”); and *b*) current exposure levels; *c*) we determined the difference between recommended and current exposure levels (“exposure difference”); *d*) we obtained the exposure response functions (ERFs) quantifying the association between exposure and mortality from the literature (Table 1); (*e*) we calculated the relative risk (RR) and (*f*) the population attributable fraction (PAF) for each exposure difference (see Supplemental Material, “UTOPHIA”).

**Table 1:** Risk estimates for all-cause mortality by exposure domain.

Exposure domain	Relative risk (95% CI)	Exposure	Age group	Study design	Reference
Physical activity^*a*^	0.81 (0.76, 0.85)	11 versus 0 MET hr/week	≥ 20 years	Meta-analysis	Woodcock et al. 2011
Air pollution^*b*^	1.07 (1.04, 1.09)	Per 10 μg/m^3^ increase in PM_2.5_ exposure	≥ 20 years	Meta-analysis	WHO Regional Office for Europe 2014b
Noise^*c*^	1.04 (1.00, 1.07)	Daytime traffic noise L_Aeq,16hr_ > 60 dB(A) versus < 55 dB(A)	≥ 25 years	Ecological study	Halonen et al. 2015
Heat^*d*^	1.19 (1.16, 1.23)	99th versus 74th temperature percentile	NA	Time-series study	Guo et al. 2014
Green space^*e*^	0.99 (0.98, 1.01)	Per 10% increase in greenness	≥ 18 years	Meta-analysis	Gascon et al. 2015
Notes: CI, confidence interval; dB(A), A-weighted average sound pressure decibel levels; L_Aeq_, A-weighted equivalent sound pressure levels in decibels; MET, metabolic equivalent of task (1 MET = 1 kcal/kg/hr); NA, not available; PM_2.5_, particulate matter with a diameter ≤ 2.5 μm. ^***a***^Mortality effect of physical activity modeled with a curvilinear exposure response function, applying a 0.25 power transformation. ^***b***^Mortality effect of air pollution modeled with a linear exposure response function. ^***c***^Mortality effect of noise modeled with a logarithmic exposure response function. ^***d***^Mortality effect of heat modeled with a linear exposure response function after determining the minimum mortality percentile (74th temperature percentile) of daily mean temperature at 21.8°C. ^***e***^Mortality effect of greenness [defined as green space surface in percent (%GS)] modeled with a linear exposure response function.					

Life expectancy and economic evaluations were also performed. We estimated average change in life expectancy based on age-specific all-cause mortality rates for Barcelona (2011) [Institut d’Estadística de Catalunya (IDESCAT) 2011] following standard life-table methods (Miller and Hurley 2003). The economic evaluation was based on the value of statistical life (VoSL) approach (3,202,968 EUR for Spain, 2012; WHO Regional Office for Europe 2014a).

### International Exposure Recommendations

***Physical activity.*** The WHO recommends that adults ≥ 18 years of age should achieve 150 min of moderate-intensity aerobic PA or 75 min of vigorous-intensity aerobic PA weekly (Table 2) (WHO 2010).

**Table 2:** Estimated premature all-cause mortality preventable in Barcelona under compliance with international exposure recommendations.

Exposure	Recommendation^*a*^	Current exposure^*b*^	Deaths (95% CI)^*c*^	Life expectancy in days (95% CI)^*d*^	Economic savings in billion € (95% CI)^*e*^
Physical activity
Adults 18–64 years	600 MET min/week	77.7 MET min/week
Adults ≥ 65 years	450 MET min/week	36.7 MET min/week	1,154 (858, 1,577)	204 (161, 259)	3.7 (2.7, 5.1)
Air pollution
Annual mean PM_2.5_	10 μg/m^3^	16.6 μg/m^3^	659 (386, 834)	52 (29, 67)	2.1 (1.2, 2.7)
Noise
Daytime (0700–2300 hours) outdoor activity noise (L_Aeq,16 hr_)	55 dB(A)	65.1 dB(A)	599 (0, 1,009)	47 (0, 81)	1.9 (0, 3.2)
Heat	Changes to urban plan may provide cooling of 4°C	> 21.8°C on 101 days (minimum mortality percentile)	376 (324, 442)	34 (29, 40)	1.2 (1.0, 1.4)
Green spaces	Access to green space ≥ 0.5 ha within 300 m linear distance	31% of residents without access to green space ≥ 0.5 ha within 300 m linear distance	116 (0, 236)	23 (0, 46)	0.4 (0, 0.8)
Total			2,904 (1,568, 4,098)	360 (219, 493)	9.3 (4.9, 13.2)
Notes: CI, confidence interval; dB(A), A-weighted average sound pressure decibel levels; L_Aeq_, A-weighted equivalent sound pressure levels in decibels; MET, metabolic equivalent of task (1 MET = 1 kcal/kg/hr); PM_2.5_, particulate matter with a diameter ≤ 2.5 μm. ^***a***^International exposure recommendation by exposure domain. ^***b***^Current exposure level in Barcelona by exposure domain (2012). ^***c***^Estimated annual premature deaths resulting from noncompliance with international exposure recommendations. ^***d***^Estimated increase in life expectancy under compliance with international exposure recommendations. ^***e***^Estimated economic savings under compliance with international exposure recommendations; based on value of statistical life (VoSL) approach (3,202,968 EUR for Spain, 2012) (WHO Regional Office for Europe 2014a).					

***Air pollution.*** Particulate matter with a diameter ≤ 2.5 μm (PM_2.5_) is a commonly used proxy for exposure to all fossil fuel combustion sources (Mueller et al. 2015). The WHO recommends that annual mean PM_2.5_ exposure concentrations should not exceed 10 μg/m^3^ (WHO 2006).

***Noise.*** The WHO recommends that daytime (0700–2300 hours) outdoor noise levels should not exceed equivalent sound pressure levels above 55 A-weighted decibels [dB(A)] (WHO 1999).

***Heat.*** Although there are no guidelines, increasing greenery and urban albedo while reducing traffic and impermeable surfaces in cities may provide cooling in the summer months by ≤ 4°C (Doick et al. 2014; Zhao et al. 2014).

***Green spaces.*** Both a European Commission working group and the WHO recommend universal access to a green space defined as living within a 300-m linear distance of a green space ≥ 0.5 ha (European Commission 2001; WHO Regional Office for Europe 2016).

### Exposure Data

***Physical activity.*** PA data were available for 3,279 Barcelona residents (*n* = 2,486, 20–64 years of age; *n* = 793, ≥ 65 years of age) through the 2011 Barcelona Health Survey, a population-based randomized sample studying the health status of Barcelona residents (Bartoll et al. 2013). PA data were extrapolated to all Barcelona residents ≥ 20 years old (Table 2).

WHO guidelines for adults 18–64 years of age/≥ 65 years of age, were translated into 600/450 metabolic equivalent of task (MET) minutes per week, respectively (see Tables S3 and S4) [International Physical Activity Questionnaire (IPAQ) 2005]. The association between PA and mortality was quantified using a curvilinear ERF, applying a 0.25 power transformation to PA (Woodcock et al. 2011). Because health benefits occur even at low levels of PA, the RR and the PAF were calculated for both the current and the recommended MET minutes per week. Estimated preventable deaths for current PA levels were subtracted from estimated preventable deaths for recommended PA levels.

Sensitivity analyses using *a*) a linear ERF and *b*) including METs accumulated by walking as part of total PA were performed (see Supplemental Material, “Physical Activity”; see also Tables S5–S8).

***Air pollution.*** Annual mean PM_2.5_ data for 2012 were available for Barcelona at the census-tract level through the European Study of Cohorts for Air Pollution Effects Land Use Regression (ESCAPE LUR) model (Eeftens et al. 2012). The exposure difference in annual mean PM_2.5_ concentrations necessary to comply with the recommendation of 10 μg/m^3^ was estimated for each census tract.

The association between PM_2.5_ and mortality was quantified using a linear ERF (WHO Regional Office for Europe 2014b). The RR and PAF corresponding to the exposure difference were calculated at the census-tract level.

Sensitivity analyses assuming achievement of *a*) the WHO interim target of an annual mean of 15 μg/m^3^ for PM_2.5_ (WHO 2006) and *b*) the lowest measured PM_2.5_ level of 5.8 μg/m^3^ were performed (see Supplemental Material, “Air Pollution”; see also Tables S9 and S10) (Krewski et al. 2009).

***Noise.*** Daytime traffic noise levels were calculated at the census-tract level using Barcelona’s strategic noise map (0700–2300 hours; L_Aeq,16hr_) (Generalitat de Catalunya 2006). The ERF for Barcelona traffic noise exposure and mortality was predicted based on available risk categories (Halonen et al. 2015), assuming a logarithmic relationship (see Supplemental Material, “Noise”; see also Table S11 and Figure S1).

The exposure difference was determined for each census tract exceeding L_Aeq,16hr_ 55 dB(A). The corresponding RR and PAF were calculated based on the predicted ERF.

As a sensitivity analysis, the PAF was calculated exclusively for the proportion of people in each census tract that self-reported noise annoyance (see Supplemental Material, “Noise”; see also Tables S12 and S13 and Figure S1) (Gobierno de España 2012).

***Heat.*** Daily mean temperatures (2009–2014) were available through a central monitor in Barcelona (Klein Tank et al. 2002). Drawing on a temperature raster map (2007, resolution 1 km) (Grupo de Investigación Kraken, Universidad Extremadura 2007) and using QGIS (v2.6.1), monthly mean temperatures were calculated at the census-tract level.

Daily mean temperatures for 2009–2014 available through the central monitor in Barcelona were averaged to obtain typical temperatures for one calendar year. Following an empirical model, the 74th daily mean temperature percentile, which defined the minimum mortality temperature percentile for Spain, was determined to be 21.8°C (see Supplemental Material, “Heat”; see also Table S14) (Guo et al. 2014). Between the 74th and 99th temperature percentiles, a linear mortality ERF was assumed. Monitor data and raster map data were combined to estimate daily mean temperatures at the census-tract level for 2011 (see Supplemental Material, “Heat”; see also Tables S14 and S15).

For days exceeding 21.8°C, the exposure difference in daily mean temperature was calculated at the census-tract level. The corresponding RR and PAF were calculated. Temperatures were theoretically reduced by 4°C, and the exposure difference was calculated for days still exceeding 21.8°C. The corresponding RR and PAF were calculated. The number of deaths attributable to temperatures reduced by 4°C was subtracted from the number of deaths attributable to the estimated temperatures for 2011.

A sensitivity analysis with a temperature reduction of 1°C was performed (see Supplemental Material, “Heat”; see also Table S16).

***Green space.*** To provide universal access to a green space ≥ 0.5 ha within a 300-m linear distance, we estimated how much green space surface (%GS) each census tract needed to have.

Green space data were available through Urban Atlas (2007, resolution 1:10,000) (European Environment Agency 2007) and the Barcelona Health Survey (Bartoll et al. 2013). Using ArcGIS, the current %GS was calculated for each census tract. Quintiles of the %GS distribution were calculated. Using GIS-derived green space data for the Barcelona Health Survey respondents (*n* = 3,417), the proportion of Health Survey respondents living within 300 m of a green space ≥ 0.5 ha was determined for each %GS quintile (see Supplemental Material, “Green Spaces”; see also Table S17). A logarithmic function was fitted to predict the %GS needed to provide universal access to a green space ≥ 0.5 ha within 300 m (see Supplemental Material, “Green Spaces”; see also Figure S2). It was predicted that each census tract would need to have 25.6%GS in order to provide universal access to a green space ≥ 0.5 ha within 300 m.

The exposure difference between the current %GS of each quintile and the necessary 25.6% was determined (see Supplemental Material, “Green Spaces”; see also Table S18). A linear ERF was used to quantify the association between green space and mortality (Gascon et al. 2016). For each exposure difference by %GS quintile, the RR and the corresponding PAF were calculated.

## Results

**Figure 2:**
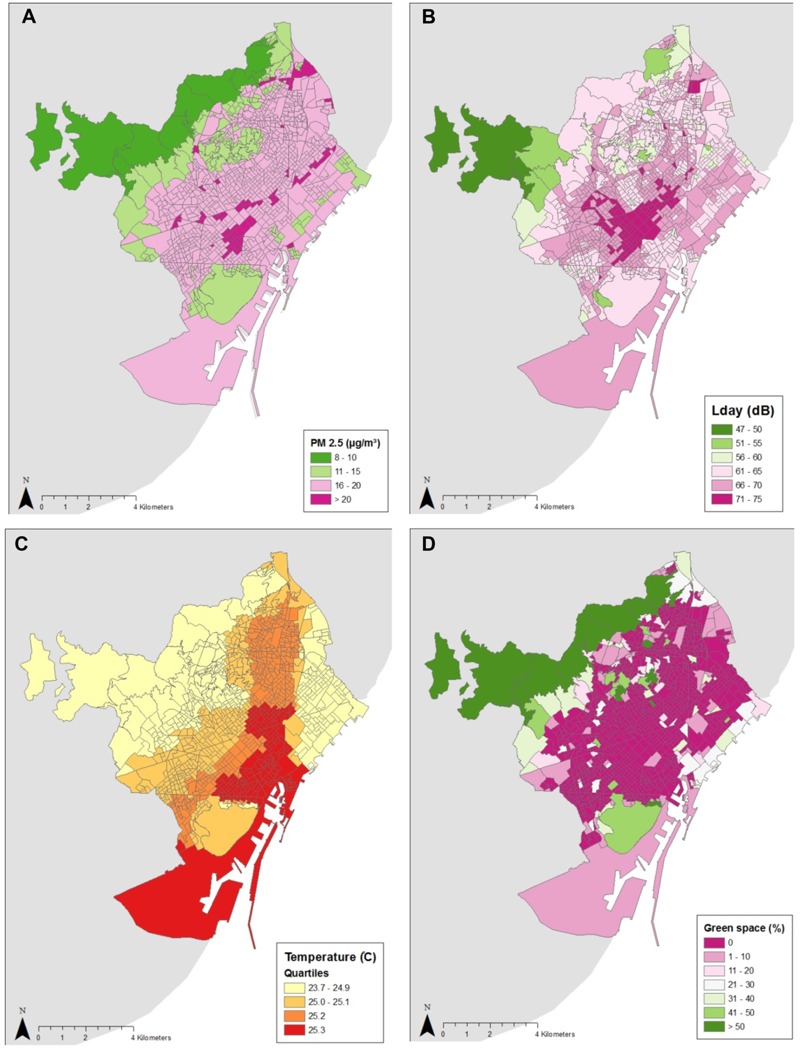
Environmental exposure maps for Barcelona at the census-tract level (*n* = 1,061). (*A*) Air pollution, PM_2.5_ annual mean; (*B*) daytime road traffic noise, L_Aeq,16hr_ (0700–2300 hours); (*C*) heat, daily mean temperature for 1 July 2011; (*D*) green spaces, green space surface in percent (GS%) of green spaces ≥ 0.5 ha. Source: Instituto Nacional de Estadística 2013. Own compilation with data taken from the INE website: www.ine.es. L_Aeq_, A-weighted equivalent sound pressure levels in decibels; PM_2.5_, particulate matter with a diameter ≤ 2.5 μm.

More than 70% of adults in Barcelona were insufficiently active (Table 2). Air pollution and traffic noise levels far exceeded the recommended values (Figure 2). Barcelona’s summer months were too hot; one third of the population did not live within the recommended distance of 300 m to a green space ≥ 0.5 ha.

**Figure 3:**
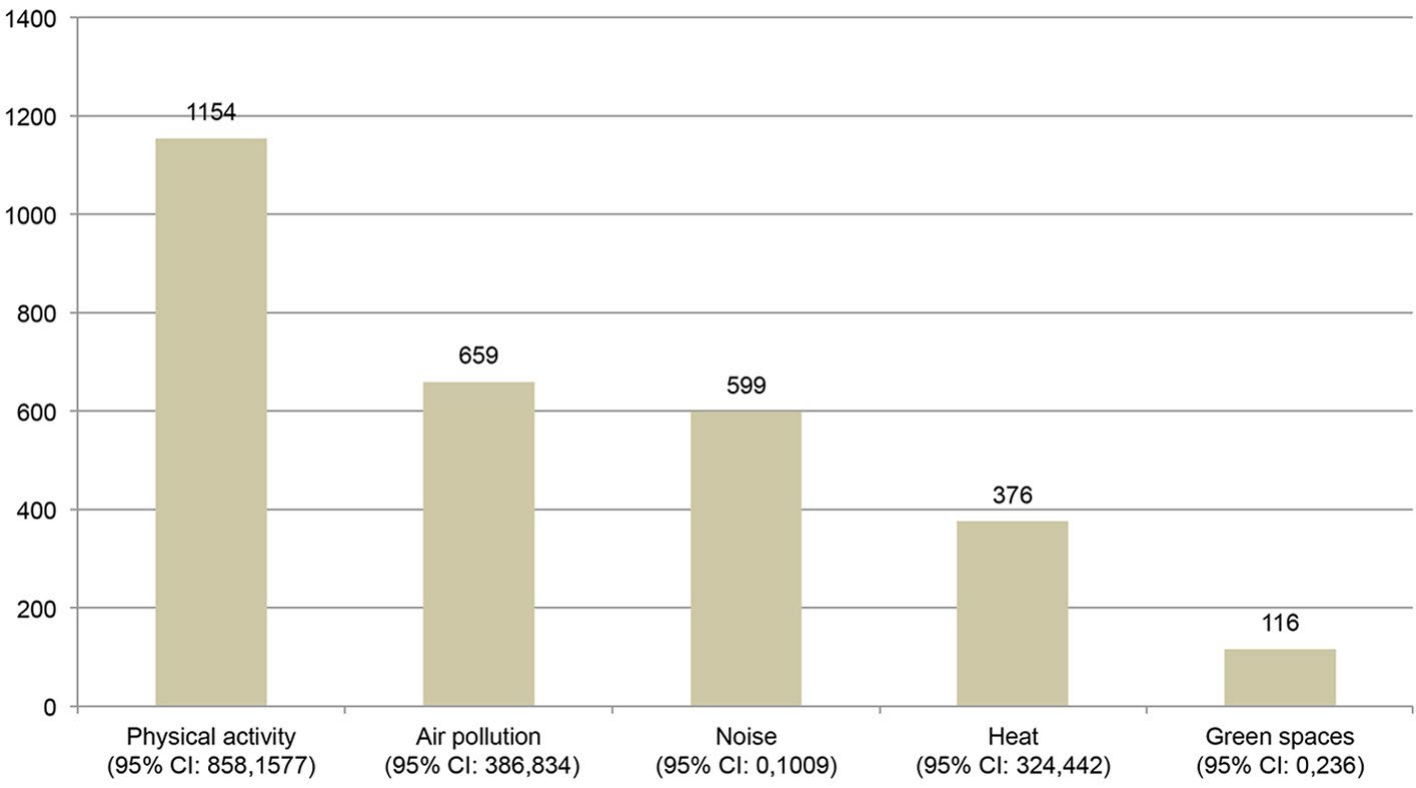
Estimated preventable deaths under compliance with exposure recommendations by exposure domain. The exposure response functions (ERF) for physical activity, air pollution and green spaces were obtained from meta-analyses. The ERF for noise was taken from an ecological study. The ERF for heat was taken from a population-level time-series study. CI, confidence interval.

Annually, 2,904 [95% confidence interval (CI): 1,568, 4,098] deaths were estimated to be preventable if Barcelona complied with international exposure recommendations (Table 2). Estimations showed that the greatest portion of preventable deaths could be attributed to increases in PA (1,154 deaths; 95% CI: 858, 1,577), followed by reductions in exposure to air pollution (659 deaths; 95% CI: 386, 834), traffic noise (599 deaths; 95% CI: 0, 1,009), and heat (376 deaths; 95% CI: 324, 442) (Figure 3). Access to a green space was estimated to have a smaller impact on mortality (116 deaths; 95% CI: 0, 236) than those of the other exposures.

Under compliance with international exposure recommendations, Barcelona’s residents were estimated to live an average of 360 (95% CI: 219, 493) days longer, and an estimated 9.3 (95% CI: 4.9, 13.2) billion EUR could be saved annually.

The results from the sensitivity analyses are presented in the Supplemental Material (“Physical Activity,” “Air Pollution,” “Noise,” “Heat,” and “Green Spaces”), and they indicate that our estimates are generally robust.

## Discussion

We developed and implemented the UTOPHIA model for Barcelona and estimated that 2,904 (i.e., nearly 20%) of all annual natural deaths in Barcelona could be prevented if international recommendations for performance of PA; exposure to air pollution, noise, and heat; and access to green space were followed. The present study is the first to quantify the effects of multiple urban and transport planning–related exposures in a city, and we showed considerable impacts of these exposures on health.

Other HIAs have estimated the impacts of some of these exposures in cities and found results comparable to ours. An HIA in Madrid, Spain, with twice as many residents and similar environmental conditions, found that a reduction of nearly 470 deaths could be attributed to a theoretical decrease in traffic noise exposure of only 1 dB(A) (Tobías et al. 2014). Other HIAs that looked at mortality effects of increases in active transport found considerable reductions in premature deaths, with most benefits attributable to increases in PA (Rojas-Rueda et al. 2011; Woodcock et al. 2014). A recent HIA for Basel, Switzerland found that expected PM_2.5_ reductions with implementation of proposed transport policy measures would result in a reduction of premature mortality by 3% (Perez et al. 2015).

### Limitations and Strengths

We have estimated that Barcelona’s compliance with international exposure recommendations would have a considerable impact on all-cause mortality. However, HIAs involve multiple assumptions that carry uncertainties in estimating health impacts and of which we could quantify only a limited extent.

The ERFs for PA, air pollution, and green spaces were obtained from the most recent meta-analyses. The evidence for the mortality effects of PA and for air pollution is stronger than for the other exposures simply because more research has been done on these exposures. The estimates of noise and green spaces are only suggestive, as indicated by the wide confidence intervals. Despite emerging evidence on green spaces providing general health benefits (Dadvand et al. 2016; Triguero-Mas et al. 2015), until now, only a few studies have investigated the association between green space and mortality. Moreover, the exposure definition of “greenness” implies uncertainties arising from heterogeneity in exposure assessment. At present, we are unaware of existing meta-analyses or quantitative reviews regarding the effects of noise and heat. The ERF for noise came from the only existing ecological study that is presently available, and the ERF for heat came from a population-level time-series study, which limits the strength of the evidence. With regard to noise, the WHO recommends that nighttime (2300–0700 hours) outdoor noise levels should not exceed equivalent sound pressure levels above 40 dB(A) (WHO 1999). However, no evidence exists on the association between nighttime noise and all-cause mortality (Halonen et al. 2015). With regard to heat, the exposure indicator used was daily mean temperature. This indicator, however, is limited in its reflection of heat stress because it does not consider other important determinants such as humidity, solar radiation or wind force.

Generally, benefit estimations are sensitive to the contextual setting and underlying population parameters. Estimations of health impacts depend largely on baseline exposure to the health pathways considered and the general health status of the population; thus, varying results can be expected in different settings. Moreover, personal choices and intrinsic motivations for behavior change (e.g., choosing to use a bicycle instead of a car), and thus exposure alterations, are unquantifiable, but they can have a large impact on health. Thus, the generalizability and causal inference of our results may be uncertain.

In addition, time lags in benefit estimations and the resulting delayed receipt of health benefits can significantly alter benefit estimations. Because we were interested in long-term effects of exposure alterations, a delay in the receipt of benefits is expected. Practical implications of this delay may be that changes to urban and transport planning practices are less relevant for younger people in terms of mortality impacts, but its importance is reinforced for older people. In times of demographic change and increasing aging populations, it is important to keep this delay in mind. In this regard, the economic impact is most likely overestimated; here, time discounting applies because benefits occurring in the future are less valuable than benefits occurring immediately.

The present study focused on mortality. Assessing the associated morbidity burden was outside the scope of this study. A further concern is the double-counting of deaths because air pollution, noise, and heat share a common source (i.e., motorized traffic) and a common mitigator (i.e., green spaces). Estimated effects might interact and synergies may exist between the exposures. At the present time, evidence of the independence of mortality effects is only available for air pollution and noise (Tétreault et al. 2013). Therefore, the results presented herein should be interpreted with caution because effect modification cannot be ruled out. Nevertheless, it is possible that we might have underestimated the air pollution burden because we only considered PM_2.5_ mortality effects. Other traffic-related air pollutants [e.g., nitrogen dioxide (NO_2_)], which we did not consider, have been shown to have independent mortality associations (Faustini et al. 2014).

The strength of this study is its novelty in terms of linking urban and transport planning–related exposures and health in an integrated way, which highlights the considerable impacts on mortality of noncompliant exposure levels. The detailed exposure data on the same spatial scale strengthen the internal validity of the study. The census-tract level and exposure models were of fairly refined resolution. The sensitivity analyses showed that our estimates were fairly robust.

Despite being unable to show the extent to which improvements of the urban environment could actually contribute towards achieving recommended exposure levels, it is believed that reconsideration of urban and transport policies would have a large impact on PA factors and environmental exposures. Therefore, HIA is a valuable tool to enhance understanding of the interrelationship between the environment and health and can assist policy makers in optimizing health gains.

### Solutions

**Figure 4:**
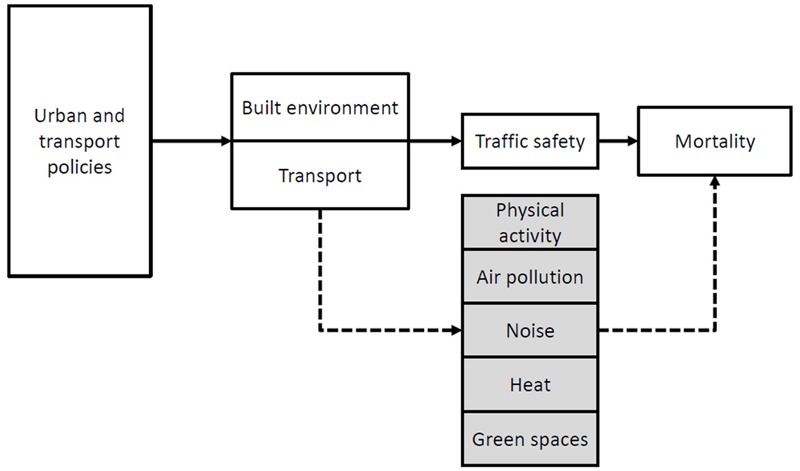
Mortality pathways of urban and transport policies. Health effects of urban and transport planning are most likely considered in terms of traffic safety. However, health pathways of physical activity, air pollution, traffic noise, heat, and green spaces show considerable impacts on natural all-cause mortality.

Solutions to the considerable burden of environmental exposures on mortality can be found, at least in part, in changes to urban and transport planning. Despite the estimated number of preventable deaths being much larger than the annual number of traffic fatalities in Barcelona (*n* = 30 for 2012) (Barcelona City Council 2013), traffic safety receives the most attention in terms of health impacts of urban and transport planning (Figure 4).

A paradigm shift in urban and transport planning is needed to provide a multidimensional approach to urban environmental quality and associated public health benefits (Brauer and Hystad 2014). Increasing public and active transport (walking and cycling for transport) while simultaneously facilitating urban greening can provide multiple health benefits.

***Physical activity.*** Insufficient PA was associated with the greatest excess mortality in Barcelona; this highlights the urgency of integrating PA into daily life. Active and public transport provide a great opportunity to do so because both forms of transport provide coincidental health gains by increases in PA. Public transport is estimated to provide an additional 10 min of walking per day (Rojas-Rueda et al. 2012), and a longitudinal study showed significant contributions of PA from active transport to overall PA as participants who increased their active transport levels had an additional 135 min of total PA per week (Sahlqvist et al. 2013).

The proportion of trips made by walking and cycling is increasing in Barcelona (increases of 0.7% and 5.6% in 2012 compared with 2011, respectively) (Barcelona City Council 2013), but further efforts are needed to reinforce these positive trends. Investment in active and public transport infrastructure and safety measures are economically justified and yield high returns (Gössling and Choi 2015).

Reinforcement of green infrastructure may also facilitate PA engagement (i.e., active transport) because exercise in green spaces is associated with higher-intensity exercising and increased enjoyment (Gladwell et al. 2013).

***Air pollution, noise, and heat.*** Exposure to air pollution, noise, and heat resulted in large contributions to the estimated mortality burden. Barcelona’s vehicle fleet of > 500,000 cars and nearly 300,000 scooters and motorcycles, and an additional daily suburban commuter fleet, result in a high volume of motorized traffic and associated emissions (Barcelona City Council 2013).

Air pollution and noise are amplified in the narrow, built-up streets typical of Barcelona owing to reduced air mass exchange within these street canyons (Marini et al. 2015) and to multiple interactions of noise waves with building facades (Van Renterghem et al. 2015). A systematic review supports our findings with the conclusion that noise and air pollution have similar but independent mortality effects (Tétreault et al. 2013).

Barcelona’s summer temperatures are reinforced by anthropogenic heat resulting from combustion by motorized traffic, reradiation by urban construction, and a shortage of green and open spaces for dissipation (Memon et al. 2008).

Key strategies for mitigating air pollution, noise, and heat are the reduction of motorized traffic by replacing it with zero- and low-emitting modes of transport (i.e., active and public transport) and the provision of urban greening. Taking opportunities with urban renewal, densely constructed gray infrastructure could be loosened up and replaced with nonradiating and green infrastructure. Vegetation can be a passive control of air pollution exposure (Abhijith and Gokhale 2015), is a natural noise barrier (Van Renterghem et al. 2015), and provides shading and cooling of the surroundings through evapotranspiration of water (Raji et al. 2015).

***Green space.*** Despite the suggested minor impact of green spaces on natural all-cause mortality, the co-benefits of PA engagement and refuge from harmful environmental exposures (i.e., air pollution, noise, and heat) make green spaces an important urban and traffic management tool.

The present study evaluated mortality effects of access to green spaces. The recommendation of a 300-m linear distance is supported by research findings suggesting that green space use declines beyond 300–400 m (Annerstedt et al. 2012). For active use (i.e., PA), however, green space attractiveness and maintenance appear to be more important than distance or size (Sugiyama et al. 2010). Furthermore, aesthetically pleasing “surrounding greenness” such as street trees or greenways may also be important and has been associated with a wide range of health indicators (Triguero-Mas et al. 2015).

Additional pathways that may help to explain the beneficial effects of green space on mortality are *a*) mitigation (of air pollution, noise, and heat) (Gascon et al. 2016); *b*) association of visual access to green spaces with stress relief, positive affect, and restoration (Wolf and Robbins 2015); *c*) improved mental health (Triguero-Mas et al. 2015); *d*) enriched biodiversity strengthening immune function (Rook 2013); and *e*) increased safety perception and social cohesion (Garvin et al. 2013; Wolf and Robbins 2015).

## Conclusions

Each year in Barcelona, nearly 20% of mortality was estimated to be attributable to noncompliance with recommended levels of PA, air pollution, noise, heat, and access to green spaces. Environmental exposures and PA factors can be modified by changes in urban and transport planning. We urge the consideration of health impacts when designing cities and emphasize the importance of *a*) the reduction of motorized traffic through promotion of active and public transport and *b*) the provision of urban greening; both of these factors can provide opportunities for PA engagement as well as mitigation of air pollution, noise, and heat.

## Supplemental Material

(522 KB) PDF
