# Supplementary material for: Urban and Transport Planning Related Exposures and Mortality: A Health Impact Assessment for Cities
Source: Environ Health Perspect. 2016 Jun 27;125(1):89–96. doi: 10.1289/EHP220 (PMC5226698; doi:10.1289/EHP220)
Supplement: (522 KB) PDF [file EHP220.s001.acco.pdf]

Environ Health Perspect DOI: 10.1289/EHP220
Advance Publication: Not Copyedited

Note to readers with disabilities: *EHP* strives to ensure that all journal content is accessible to all readers. However, some figures and Supplemental Material published in *EHP* articles may not conform to 508 standards due to the complexity of the information being presented. If you need assistance accessing journal content, please contact ehp508@niehs.nih.gov. Our staff will work with you to assess and meet your accessibility needs within 3 working days.

Supplemental Material

Urban and Transport Planning Related Exposures and Mortality: A Health Impact Assessment for Cities

Natalie Mueller, David Rojas-Rueda, Xavier Basagaña, Marta Cirach, Tom Cole-Hunter, Payam Dadvand, David Donaire-Gonzalez, Maria Foraster, Mireia Gascon, David Martinez, Cathryn Tonne, Margarita Triguero-Mas, Antònia Valentín, and Mark Nieuwenhuijsen

Table of Contents

Supplemental Material A. UTOPHIA

Table S1. Barcelona natural all-cause mortality rate for population ≥20 years in 2012

Table S2. Risk estimates for all-cause mortality by exposure domain

Supplemental Material B. Physical activity

Table S3. Physical activity distribution of ‘insufficiently-active’ Barcelona Health Survey respondents 20-64 years (N=1,835)

Table S4. Physical activity distribution of ‘insufficiently-active’ Barcelona Health Survey respondents ≥65 years (N=643)

Table S5. Sensitivity analysis. Linear exposure response function for physical activity and mortality

Table S6. Sensitivity analysis. Physical activity distribution of 'insufficiently-active' Barcelona Health Survey respondents 20-64 years considering MET minutes/ week accumulated by walking (METs = 3)

Table S7. Sensitivity analysis. Physical activity distribution of 'insufficiently-active' Barcelona Health Survey respondents ≥65 years considering MET minutes/ week accumulated by walking (METs = 2.5)

Table S8. Sensitivity analysis. Estimated preventable deaths for physical activity considering MET minutes/ week accumulated by walking

Supplemental Material C. Air pollution

Table S9. Sensitivity analysis. Estimated preventable deaths under compliance with Interim-Target 3 of 15 μg/m^3 $PM_{2.5}$ as defined by WHO air quality guidelines

Table S10. Sensitivity analysis. Estimated preventable deaths under compliance with lowest measured $PM_{2.5}$ level of 5.8 μg/m^3

Supplemental Material D. Noise

Table S11. All-cause mortality risks associated with traffic noise exposure levels

Figure S1. Exposure response function for traffic noise and all-cause mortality in Barcelona

Table S12. Self-reported noise annoyance available through Indicadores de Vulnerabilidad Urbana on census tract level

Table S13. Sensitivity analysis. Estimated attributable deaths to self-reported noise annoyance rate across census tracts

Supplemental Material E. Heat

Table S14. Minimum mortality temperature percentile of daily mean temperature for Barcelona (2009-2014)

Table S15. Estimated mortality impact of exceeding the 74th daily mean temperature percentile after decreasing urban temperatures 4 ºC

Table S16. Sensitivity analysis. Estimated mortality impact of exceeding the 74th daily mean temperature percentile after decreasing urban temperatures 1 ºC

Supplemental Material F. Green spaces

Table S17. Correlation of %GS of the census tracts and Barcelona Health Survey respondents living within 300 m linear distance to a green space of ≥0.5ha

Figure S2. Logarithmic function between %GS and the proportion of Barcelona residents with access to a green space ≥0.5 ha within 300 m linear distance

Table S18. Necessary increase in %GS for each quintile in order to provide universal access to a green space ≥0.5 ha within 300 m linear distance

References

SUPPLEMENTAL MATERIAL A. UTOPHIA

The Urban and TranspOrt Planning Health Impact Assessment (UTOPHIA) tool was developed following quantitative, environmental health impact assessment (HIA) (World Health Organization 2015b). The 2012 Barcelona mortality rate was obtained through the Barcelona Public Health Agency (Agència de Salut Pública de Barcelona 2012). All external causes of deaths were excluded (Table S1).

Table S1. Barcelona natural all-cause mortality rate for population ≥20 years in 2012

	Deaths	Mortality rate per 100,000	Population ≥20 years[b]	Year	Reference
Natural all-cause mortality[a]	15,049	1,108	1,357,361	2012	Agència de Salut Pública

[a] excluding external causes of death (i.e. accidents, homicides, suicides)
[b] Barcelona total population (all ages) in 2012 N=1,620,943

Exposure response functions (ERF) for physical activity, air pollution, noise, heat and green spaces were obtained from the literature, based on best available evidence (Table S2).

Table S2. Risk estimates for all-cause mortality by exposure domain

Exposure domain	Relative Risk (95% CI)	Exposure	Age group	Study design	Reference
Physical activity[a]	0.81 (0.76-0.85)	11 vs 0 MET hours/ week	≥20 years	Meta-analysis	Woodcock et al. 2011
Air pollution[b]	1.07 (1.04-1.09)	per 10 µg/m^3 increase in $PM_{2.5}$ exposure	≥20 years	Meta-analysis	WHO 2014
Noise[c]	1.04 (1.00-1.07)	Day time traffic noise $L_{Aeq,16hr}$ >60 dB(A) vs <55 dB(A)	≥25years	Ecological study	Halonen et al. 2015
Heat[d]	1.19 (1.16-1.23)	99th vs 74th temperature percentile	NA	Time-series study	Guo et al. 2014
Green space[e]	0.99 (0.98-1.01)	per 10% increase in greenness	≥18 years	Meta-analysis	Gascon et al. 2015

CVD=cardiovascular disease; dB(A)=A-weighted average sound pressure decibel levels; MET=metabolic equivalent of task (1 MET=1 kcal * kg^{-1} * h^{-1}); NA=not available; $PM_{2.5}$=particulate matter ≤2.5 µg; 95% CI=95% confidence interval.

[a] Mortality effect of physical activity modeled with a curvilinear exposure response function, applying a 0.25 power transformation.

[b] Mortality effect of air pollution modeled with a linear exposure response function.

[c] Mortality effect of noise modeled with a logarithmic exposure response function.

[d] Mortality effect of heat modeled with a linear exposure response function, after determining the minimum mortality percentile (74th temperature percentile) of daily mean temperature at 21.8 ºC.

[e] Mortality effect of greenness (defined as green space surface in % (%GS)) modeled with a linear exposure response function.

We calculated the relative risk (RR) for mortality for the 'exposure difference' between the recommended exposure level (counterfactual exposure) and the current exposure level:

$$RR_{exposure\ difference} = \exp(((\ln(RR))/exposure_{current})*(exposure_{recommended}))$$

In order to calculate the $RR_{exposure\ difference}$, we took the logarithm to the base of e (ln) of the RR and we adjusted the ln(RR) to the exposure difference. In order to take the 'anti-log', the ln(RR) was exponentiated. The exponentiated ln(RR) is the RR corresponding to the exposure difference.

Example: Air pollution census tract # 0101001001

RR=1.07 per 10 µg/m^3 $PM_{2.5}$ (World Health Organization 2014)

# Census tract	Recommended annual mean $PM_{2.5}$ µg/m^3	Current annual mean $PM_{2.5}$ µg/m^3	Exposure difference µg/m^3	Relative Risk
0101001001	10 µg/m^3	17.77 µg/m^3	17.77 µg/m^3 − 10 µg/m^3=7.77 µg/m^3	?

$$RR_{exposure\ difference} = \exp(((\ln(1.07))/10)*7.77) = 1.05$$

We calculated the population attributable fraction (PAF) for each 'exposure difference' the following:

$$\text{PAF} = \frac{\sum_{i=1}^{n} \text{Pi RRi} - \sum_{i=1}^{n} \text{P'i RRi}}{\sum_{i=1}^{n} \text{Pi RRi}}$$

Pi = proportion of population at exposure level i, current exposure

P'i = proportion of population at exposure level i, recommended level of exposure

RR = the relative risk at exposure level i

n = the number of exposure levels

The PAF is the proportional reduction in mortality that would occur if exposure to the risk factor (i.e. physical activity, air pollution, noise, heat, green spaces) was reduced or increased to an alternative ideal exposure scenario (i.e. international exposure recommendation) (World Health Organization 2015a).

**# Census tract**	**Annual mean $PM_{2.5}$ µg/m^3**	**Exposure difference µg/m^3**	**Relative Risk**
0101001001	17.77 µg/m^3	7.77 µg/m^3	1.05

PAF= (RR-1)/RR

PAF=(1.05-1)/1.05

PAF=0.05

Estimated number of deaths attributable to excess $PM_{2.5}$ exposure = PAF*expected mortality rate

**# Census tract**	**Population**	**Expected mortality**<br><br>**Barcelona mortality rate: 1,108/ 100,000**	**Estimated number of deaths attributable to $PM_{2.5}$ in Census tract 0101001001**
0101001001	1,195	1,195*(1,108/100,000)=13.24	13.24*0.05=0.67

In census tract # 0101001001 0.67 deaths are estimated to be attributable to the excess $PM_{2.5}$ annual mean exposure of 7.77 $\mu g/m^3$.

SUPPLEMENTAL MATERIAL B. Physical activity

Physical activity (PA) data were available for 3,279 Barcelona residents ≥20 years through a modified version of the IPAQ-short questionnaire included in the 2011 Barcelona Health Survey (N=2,486 20-64 years; N=793 ≥65 years). The Barcelona Health Survey is a population-based randomized sample studying the health status of Barcelona residents (Bartoll et al. 2013).

We translated WHO PA guidelines for adults 18-64 years, recommending 150 minutes moderate-intensity aerobic PA or 75 minutes of vigorous-intensity aerobic PA weekly, into 600 metabolic equivalent of task (MET) minutes/ week, according to the Guidelines for Data Processing and Analysis of the IPAQ-short (moderate-intensity aerobic PA=4 METs; vigorous-intensity aerobic PA=8 METs) (Table S3) (IPAQ Webpage 2005).

METs express energy expenditures during PA. 1 MET is considered the resting metabolic rate (RMR) and is the energy cost of a person at rest (Ainsworth et al. 2011). The RMR is defined as 1.0 kcal * kg^{-1} * h^{-1}.

The proportion of Barcelona Health Survey respondents 20-64 years (N=2,486) currently complying with 600 MET minutes/ week was determined.

651 Barcelona Health Survey respondents complied with 600 MET minutes/ week = 26%.

1,835 Barcelona Health Survey respondents did not comply with 600 MET minutes/ week = 74% (considered ‘insufficiently active’).

Table S3. Physical activity distribution of ‘insufficiently-active’ Barcelona Health Survey respondents 20-64 years (N=1,835)

**Age groups**	**# insufficiently-active Health Survey respondents**	**%**	**Average MET minutes/ week achieved of moderate to vigorous intensity aerobic PA**	**Average MET minutes/ week needed to achieve 600 MET minutes/ week**
20-24 years	114	6.2	91.58	508.42
25-29 years	171	9.3	90.178	509.82
30-34 years	249	13.6	82.67	517.33
35-39 years	249	13.6	85.70	514.30
40-44 years	246	13.4	88.68	511.32
45-49 years	234	12.8	57.09	542.91
50-54 years	204	11.1	72.70	523.15
55-59 years	178	9.7	76.85	523.15
60-64 years	190	10.4	57.05	542.95
Total	1,835	100		

MET=metabolic equivalent of task; PA=physical activity

For older adults, moderate and vigorous-intensity aerobic activity involves a moderate and vigorous level of effort relative to an individual's aerobic fitness (Nelson et al. 2007). Therefore, we translated the WHO guideline for adults ≥65 years into 450 MET minutes/ week (moderate-intensity aerobic PA=3 METs; vigorous-intensity aerobic PA=6 METs) (Table S4) (IPAQ Webpage 2005).

The proportion of Barcelona Health Survey respondents ≥65 years (N=793) currently complying with 450 MET minutes/ week was determined.

150 Barcelona Health Survey respondents complied with 450 MET minutes/ week = 19%.

643 Barcelona Health Survey respondents did not comply with 450 MET minutes/ week = 81% (considered 'insufficiently active').

Table S4. Physical activity distribution of 'insufficiently-active' Barcelona Health Survey respondents ≥65 years (N=643)

Age groups	# insufficiently-active Health Survey respondents	%	Average MET minutes/ week achieved of moderate to vigorous intensity aerobic PA	Average MET minutes/ week needed to achieve 450 MET minutes/ week
65-69 years	157	24.4	39.13	410.87
70-74 years	124	19.3	32.18	417.82
75-79 years	135	21.0	27.33	422.67
80-84 years	125	19.4	14.64	435.36
85-89 years	75	11.7	19.00	431.00
90-94 years	25	3.9	19.80	430.20
95-99 years	2	0.3	105.00	345.00
Total	643	100		

MET=metabolic equivalent of task; PA=physical activity

As already benefits occur at low levels of PA, the RR and PAF were calculated for both the current and the recommended MET minutes/ week. We modeled the mortality impacts of PA with a curvilinear ERF applying a 0.25 power transformation for PA, as measured in MET hours/ week (Woodcock et al. 2011). Estimated preventable deaths for current PA levels were subtracted from estimated preventable deaths for recommended PA levels.

Example: Physical activity of age group 20-24 years

Current MET minutes/ week = 91.57

Current MET hours/ week = 1.51 (91.57/ 60)

Recommended MET minutes/ week = 600

Recommended MET hours/ week = 10 (600/ 60)

Mortality RR = 0.81 per 11 MET hours/ week (Woodcock et al. 2011)

Mortality risk for current MET hours/ week

RR=EXP(LN(0.81)*((1.51/11)^(0.25)))

RR= 0.89

Mortality risk for recommended MET hours/ week

RR=EXP(LN(0.81)*((10/11)^(0.25)))

RR= 0.81

Sensitivity analyses for physical activity

(1) We conducted a sensitivity analysis using a linear exposure response function (ERF) for physical activity and mortality (Table S5).

Table S5. Sensitivity analysis. Linear exposure response function for physical activity and mortality

**Exposure**	**Recommendation**	**Estimated preventable deaths (95% CI)**
Physical activity		
Adults 18-64 years	600 MET minutes/ week	1511 (1142-2019)
Adults ≥65 years	450 MET minutes/ week	440 (334-585)
Total		1951 (1476-2604)

MET=metabolic equivalent of task

(2) We conducted a sensitivity analysis considering the MET minutes/ week accumulated by Barcelona's residents through walking as part of total PA, as walking is common in Barcelona.

(Adults 20-64 years walking = 3 METs; Adults ≥65 years walking = 2.5 METs) (Table S6 and Table S7).

The proportion of adults 20-64 years (N=2,486) complying with 600 MET minutes/ week considering the METs accumulated by walking as part of total PA was determined (Table S6).

1,721 Barcelona Health Survey respondents complied with 600 MET minutes/ week = 70%.

764 Barcelona Health Survey respondents did not comply with 600 MET minutes/ week = 30%.

The proportion of adults ≥65 years (N=793) complying with 450 MET minutes/ week considering the METs accumulated by walking as part of total PA was determined (Table S7).

548 Barcelona Health Survey respondents complied with 450 MET minutes/ week = 70%.

245 Barcelona Health Survey respondents did not comply with 450 MET minutes/ week = 30%.

Table S6. Sensitivity analysis. Physical activity distribution of 'insufficiently-active' Barcelona Health Survey respondents 20-64 years considering MET minutes/ week accumulated by walking (METs = 3)

Age groups	# insufficiently-active Health Survey respondents	%	Average MET minutes/ week achieved	Average MET minutes/ week needed to achieve 600 MET minutes/ week
20-24 years	39	5.1	256.42	343.58
25-29 years	79	10.3	323.17	276.83
30-34 years	103	13.5	323.56	276.44
35-39 years	109	14.3	312.28	287.72
40-44 years	95	12.4	280.97	319.03
45-49 years	107	14.0	279.69	320.31
50-54 years	79	10.3	278.427	321.58
55-59 years	87	11.4	299.86	300.14
60-64 years	66	8.6	302.70	297.30
Total	764	100		

MET=metabolic equivalent of task; PA=physical activity

Table S7. Sensitivity analysis. Physical activity distribution of 'insufficiently-active' Barcelona Health Survey respondents ≥65 years considering MET minutes/ week accumulated by walking (METs = 2.5)

Age groups	# insufficiently-active Health Survey respondents	%	Average MET minutes/ week achieved	Average MET minutes/ week needed to achieve 450 MET minutes/ week
65-69	52	21.2	220.43	229.57
70-74	32	13.1	193.67	256.33
75-79	47	19.2	140.90	309.10
80-84	59	24.1	171.82	278.18
85-89	39	15.9	150.71	299.29
90-94	15	6.1	131.00	319.00
95-99	1	0.4	175.00	275.00
Total	245	100		

MET=metabolic equivalent of task; PA=physical activity

We estimated that when considering the METs accumulated by walking as part of total PA, the PA distribution of Barcelona residents changed considerably. Few MET minutes/ week appear to be accumulated by moderate and vigorous-intensity PA.

Table S8. Sensitivity analysis. Estimated preventable deaths for physical activity considering MET minutes/ week accumulated by walking

Exposure	Recommendation	Current exposure	Estimated preventable deaths (95% CI)
Physical activity			
Adults 18-64 years	600 MET minutes/week	295.23 MET minutes/week	138 (102-191)
Adults ≥65 years	450 MET minutes/week	169.08 MET minutes/ week	57 (42-79)
Total			195 (144-270)

We estimated that when considering the MET minutes/ week accumulated by walking as part of total PA, annually 195 premature deaths could be prevented in Barcelona.

SUPPLEMENTAL MATERIAL C. Air pollution

Sensitivity analyses for air pollution

We conducted sensitivity analyses for $PM_{2.5}$ assessing the mortality impact (1) if Barcelona complied with the Interim-Target 3 (IT-3) of 15 μg/m^3 $PM_{2.5}$ as this is assumed an achievable goal Barcelona could comply with, if appropriate measures were taken (Table S9) (World Health Organization 2006); and (2) if Barcelona was able to comply with the lowest measured $PM_{2.5}$ level of 5.8 μg/m^3 (Table S10) (Krewski et al. 2009).

Table S9. Sensitivity analysis. Estimated preventable deaths under compliance with Interim-Target 3 of 15 μg/m^3 $PM_{2.5}$ as defined by WHO air quality guidelines

**Exposure**	**Recommendation**[a]	**Current exposure**	**Estimated preventable deaths (95% CI)**
Annual mean $PM_{2.5}$	15 μg/m^3	16.61 μg/m^3	191 (111-242)

[a] WHO air quality guidelines and interim targets for particulate matter: annual mean concentrations

Table S10. Sensitivity analysis. Estimated preventable deaths under compliance with lowest measured $PM_{2.5}$ level of 5.8 µg/m^3

**Exposure**	**Recommendation** [a]	**Current exposure**	**Estimated preventable deaths (95% CI)**
Annual mean $PM_{2.5}$	5.8 µg/m^3	16.61 µg/m^3	1060 (624-1337)

[a] WHO air quality guidelines and interim targets for particulate matter: annual mean concentrations

SUPPLEMENTAL MATERIAL D. Noise

Road traffic noise was available through Barcelona's strategic noise map (Generalitat de Catalunya 2006). Using ArcGIS (v10.0) traffic noise data was available weighted by road length for each census tract (N=1,061).

Noise indicators:

As provided by Directive 2002/49/EC relating to the assessment and management of environmental noise, the day is 12 hours, the evening four hours and the night eight hours, However, EU Member states may shorten the evening period by one or two hours and lengthen the day and/ or the night period accordingly.

Traffic noise data weighted by road length was available for day time (14 hr; 7:00-21:00 hr) and evening time (2 hr; 21:00-23:00 hr).

Noise_m_diurnal = 14 hr

Noise_m_evening = 2 hr

For our analysis we used the noise indicator $L_{Aqe,16hr}$ as also used by Halonen et al. 2015.

To retrieve road traffic noise $L_{Aqe,16hr}$ following formula was applied:

$$L_{Aqe,16hr} = 10 \log_{10} \left(\frac{1}{16} \left(14 \times 10^{\frac{Lday}{10}} + 2 \times 10^{\frac{Lev}{10}}\right)\right)$$

Barcelona daily mean $L_{Aqe,16hr}$ = 65.1 dB(A)

The ERF for Barcelona traffic noise exposure and mortality was predicted based on available risk categories (Table S11) (Halonen et al. 2015), assuming a logarithmic relationship (R^2=0.93) (Figure S1).

Table S11. All-cause mortality risks associated with traffic noise exposure levels

Noise dB(A) categories[a] $L_{Aeq,16hrs}$	Traffic noise mean exposure level dB(A) in Barcelona	N census tracts (total=1,061)	Relative Risk[b]	95% lower CI	95% upper CI
<55 dB(A)	51.51	11	1.00		
55-60 dB(A)	58.33	63	1.03	1.01	1.05
>60 dB(A)	65.65	987	1.04	1	1.07

RR=relative risk
[a] Noise dB(A) categories were derived from Halonen et al. 2015
[b] Risk estimates were derived from Halonen et al. 2015

Figure S1. Exposure response function for traffic noise and all-cause mortality in Barcelona

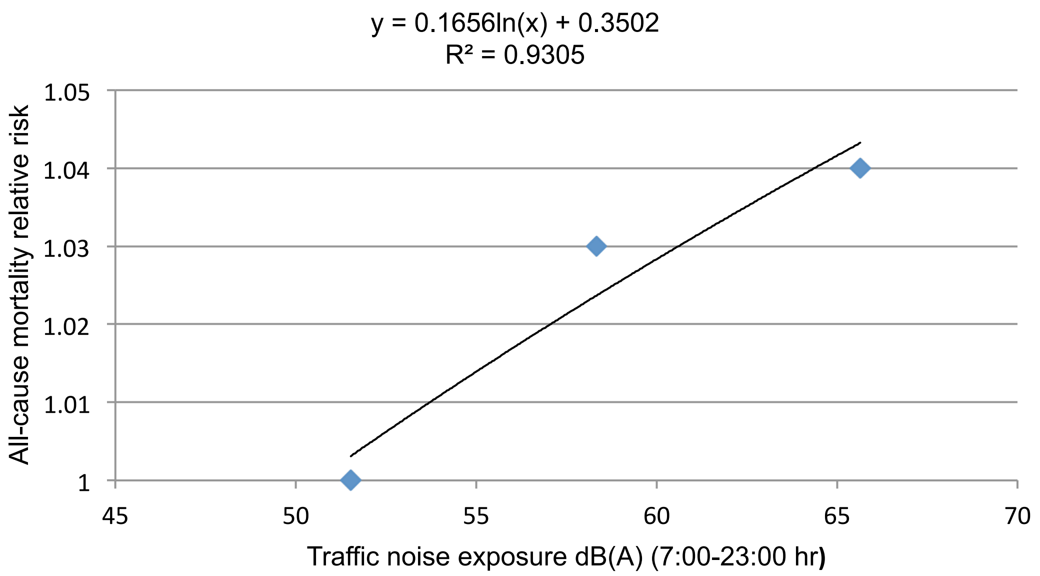

We calculated the ERF for the association between $L_{Aqe,16hr}$ day time traffic noise exposure dB(A) (7:00-23:00 hr) and all-cause mortality in Barcelona. We calculated the RR for $L_{Aqe,16hr}$ for each census tract:

$RR_{census\ tract} = 0.1656*\ln(L_{Aqe,16hr;\ census\ tract}) + 0.3502$

We calculated the PAF for each $RR_{census\ tract}$.

Sensitivity analyses for noise

In contrast to air pollution, noise may expose directional with large exposure level differences depending on location. Therefore, as a sensitivity analysis, instead of considering the entire population ≥20 years living in each census tract, the PAF was calculated exclusively for the proportion of people self-reporting annoyance from noise. Self-reported noise annoyance was available through the Indicadores de Vulnerabilidad Urbana (Table S12). (Gobierno de España 2012). It was estimated that on average 43.9% of Barcelona residents feel annoyed by traffic noise and therefore possibly vulnerable to mortality effects. (Table S13).

Table S12. Self-reported noise annoyance available through Indicadores de Vulnerabilidad Urbana on census tract level

**Road traffic noise categories in dB(A)[a]**	**Mean dB(A) in Barcelona**	**N census tracts (total=1,061)**	**Proportion of people reporting annoyance (%)**
<55 dB(A)	51.52	11	31.49
55-60 dB(A)	58.33	63	34.32
>60 dB(A)	65.65	987	44.66

[a] Categories derived from Halonen et al. 2015

Table S13. Sensitivity analysis. Estimated attributable deaths to self-reported noise annoyance rate across census tracts

Mean self-reported noise annoyance across census tracts (N=1,061)	Estimated attributable deaths (95% CI)
43.9%	272 (0-458)

Self-reported noise annoyance data on census tract level was available through the Indicadores de Vulnerabilidad Urbana.

SUPPLEMENTAL MATERIAL E. Heat

Daily mean temperature (2009-2014) data were available through a central monitor in Barcelona located at Zona Universitària (Klein Tank 2002). Through a temperature raster map, monthly minimum and maximum temperature data were available on census tract level for 2007 (raster resolution: 1km) (Grupo de Investigación Kraken. Universidad Extremadura 2007). Using QGIS (v2.6.1) and the temperature raster map, we were able to calculate monthly mean temperatures on census tract level.

Daily mean temperature (2009-2014) obtained by the monitor were averaged to obtain typical temperatures for one calendar year. Following an empirical model, the 74th and 99th daily mean temperature percentiles were determined following an empirical method (Table S14) (Guo et al. 2014). The 74th daily mean temperature percentile was previously defined as the 'minimum mortality temperature percentile' for Spain (Guo et al. 2014). For Barcelona the 74th percentile was determined at 21.8 ºC.

As heat effects estimated for Spain by Guo et al. 2014, appear to be following a close to linear relationship between the 74th and 99th daily mean temperature percentiles, a linear ERF for mortality was assumed. The temperature difference between the 74th and 99th temperature percentile was 3.4 ºC for Barcelona.

Table S14. Minimum mortality temperature percentile of daily mean temperature for Barcelona (2009-2014)

Mean temperature (2009-2014)	74th temperature percentile (minimum mortality percentile)	99th temperature percentile	Temperature difference 74th-99th temperature percentile
16.6 ºC	21.8 ºC	25.7 ºC	3.4ºC

To derive daily mean temperature on census tract level (unit of ERF), daily mean temperature levels for 2011 measured at the central monitor were combined with the QGIS derived monthly mean temperature data on census tract level for 2007 according to the following formula:

Daily mean temperature on census tract level = (Daily mean temperature measurement station * GIS monthly average temperature on census tract level (temperature raster map)) / Monthly average temperature measurement station

During 2011, for those days exceeding 21.8 ºC daily mean temperature, the ‘exposure difference’ was calculated for each census tract. The corresponding RR and PAF were calculated.

As daily mortality was not available, it was assumed that people die with the same rate over every day:

1,108/365 =3.03 deaths/ 100,000 people

The previous steps were repeated with the number of days still exceeding the threshold of 21.8 ºC, after theoretically decreasing temperatures 4 ºC. The number of deaths estimated to be attributable to daily mean temperatures reduced by 4 ºC was subtracted from the number of deaths previously estimated to be attributable to actually measured temperatures in 2011 (Table S15).

Table S15. Estimated mortality impact of exceeding the 74th daily mean temperature percentile after decreasing urban temperatures 4 ºC

		**Number of days exceeding 21.8 ºC daily mean temperature (‘minimum mortality percentile’)**	**Estimated attributable heat deaths (95% CI)[b]**
**Daily mean temperature (2011)[a]**	16.1 ºC	101 (May – September)	389 (335-457)
**Theoretical 4 ºC decrease in daily mean temperature (2011)**	12.1 ºC	11 (June – August)	12 (11-15)
**Total**			376 (324-442)

[a] Data obtained by combining 2011 data from European Climate Assessment & Dataset (ECA&D) obtained at centrally located measurement station (Zona Universitària) and a QGIS derived temperature raster map of 2007.
[b] As daily natural-cause mortality incidence was not available, it was assumed that people die with the same rate over 365 days; (1,108 deaths/ 100,000 person)/ 365 days =3.03 deaths/ 100,000 persons.

Sensitivity analysis for heat

(1) A sensitivity analysis was conducted assuming a decrease of daily mean temperature by 1 ºC, as this appears a more realistic scenario and interim goal Barcelona could aim to achieve with appropriate measures taken (Table S16).

Table S16. Sensitivity analysis. Estimated mortality impact of exceeding the 74th daily mean temperature percentile after decreasing urban temperatures 1ºC

		**Number of days exceeding 21.8 ºC daily mean temperature ('minimum mortality percentile')**	**Estimated attributable heat deaths (95% CI)[b]**
**Daily mean temperature (2011)[a]**	16.1 ºC	101 (May – September)	389 (335-457)
**Theoretical 1 ºC decrease in daily mean temperature (2011)**	15.1 ºC	84 (May – September)	239 (205-281)
**Total**			150 (129-175)

[a] Data obtained by combining 2011 data from European Climate Assessment & Dataset (ECA&D) obtained at centrally located measurement station (Zona Universitària) and a QGIS derived temperature raster map of 2007.
[b] As daily natural-cause mortality incidence was not available, it was assumed that people die with the same rate over 365 days; (1,108 deaths/ 100,000 person)/ 365 days =3.03 deaths/ 100,000 persons.

SUPPLEMENTAL MATERIAL F. Green spaces

In order to provide universal access to a green space ≥0.5 ha within 300 m linear distance, we estimated how much green space surface (%GS) each census tract needs to have.

Using Urban Atlas (European Environment Agency 2007) and ArcGIS (v10.0), the percentage of green space surface (%GS) of green spaces ≥0.5 ha were calculated for each census tract. Quintiles of %GS were calculated among the census tracts (Table S17).

GIS derived data of access to a green space ≥0.5 ha within 300 m linear distance were available for 3,417 Barcelona Health Survey respondents (‘yes/’no’) (Table S17). These 3,417 Barcelona Health Survey respondents were matched to their corresponding %GS quintile by matching census tract numbers. Barcelona Health Survey respondents were assumed representative for the entire Barcelona population (2012). With ascending %GS quintile an increasing number of Barcelona Health Survey respondents was estimated to have access to a green space ≥0.5 ha within 300 m linear distance (‘yes’) (Table S17).

Table S17. Correlation of %GS of the census tracts and Barcelona Health Survey respondents living within 300 m linear distance to a green space of ≥0.5ha

**Urban Atlas (ArcGIS)**			**2011 Barcelona Health Survey respondents (ArcGIS)**						
% green space surface of census tracts (%GS)			Distribution of Barcelona Health Survey respondents among quintiles[a]		Barcelona Health Survey respondents with 'access to a green space ≥0.5ha within 300m linear distance'				Population affected
Quintiles	Mean	Maximum (Quintile cut-off)	# Barcelona Health Survey respondents	%	# 'Yes'	# 'No'	# Total	% of people with access to green space	Proportion of people that still needs access to achieve 100% access to green space ≥0.5ha within 300 m
1st quintile	0.00	0	781	22.9	227	554	781	29.07	70.93
2nd quintile	0.27	1.10	714	20.9	419	295	714	58.68	41.31
3rd quintile	2.18	3.47	682	20	514	168	682	75.37	24.63
4th quintile	5.11	7.62	584	17.1	514	70	584	88.01	11.99
5th quintile	18.22	81.83	656	19.2	630	26	656	96.04	3.96
	25.6		3,417	100	2,304	1,113	3,417	100	

%GS= green space surface in %
[a]Barcelona Health Survey respondents were matched by census tract number to their %GS quintile.

The proportion of Barcelona Health Survey respondents having access to a green space ≥0.5 ha within 300 m linear distance (‘yes’) was correlated with the corresponding %GS quintiles, by fitting a logarithmic function (R^2=0.98) (Figure S2). For 100% of Barcelona Health Survey respondents to have access to a green space ≥0.5 ha within 300 m linear distance (‘yes’), the corresponding %GS was predicted (Figure S2).

$y=9.1271*\ln(x) + 70.388$

y=100% access to a green space ≥0.5 ha within 300 m linear distance

x=?

$x=\exp((100-70.388)/9.1271)$

x=25.6%

It was predicted that if 25.6% of each census tract was covered with green space, 100% of Barcelona’s population would have access to a green space ≥0.5 ha within 300 m linear distance.

Figure S2. Logarithmic function between %GS and the proportion of Barcelona residents with access to a green space ≥0.5 ha within 300 m linear distance

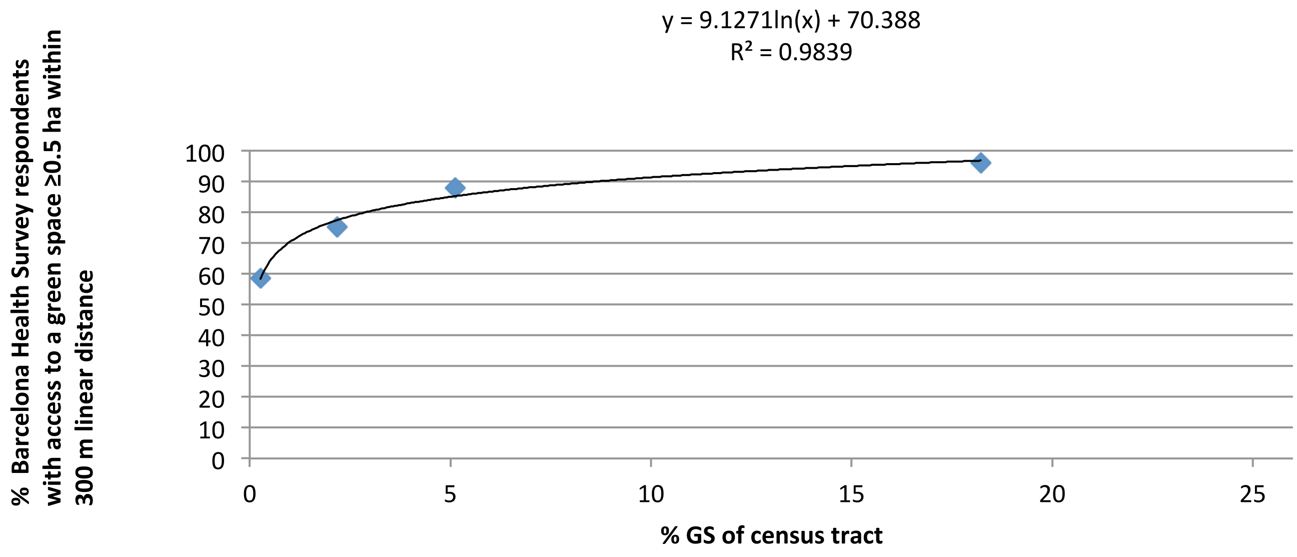

%GS=green space surface in %.

The %GS mean for each quintile (Table S17) was subtracted from the predicted 25.6 %GS needed to provide universal access (Table S18). This 'exposure difference' was the estimated increase in %GS necessary for each quintile in order to provide 100% of Barcelona residents with access to a green space ≥0.5 ha within 300 m linear distance and was used as the exposure in the linearly modeled ERF (per

10% increase in greenness) for mortality (Gascon et al. 2016). The RR and the corresponding PAF were calculated for the 'exposure difference' for each %GS quintile.

Table S18. Necessary increase in %GS for each quintile in order to provide universal access to a green space ≥0.5 ha within 300 m linear distance

**%GS quintiles**	**Current %GS mean of census tracts**	**Necessary increase in %GS to provide 100% of Barcelona population with access to a green space ≥0.5 ha within 300 m linear distance**
1st quintile	0.00	25.65
2nd quintile	0.27	25.38
3rd quintile	2.18	23.46
4th quintile	5.11	20.53
5th quintile	18.22	7.43

%GS=green space surface in %.

REFERENCES

Agència de Salut Pública de Barcelona. 2012. Llibre Mortalitat Anual. Barcelona 2012, homes i dones. Taxes de mortalitat de les primeres 15 causes de mort. Available: http://www.aspb.cat/quefem/sisalut/SISalutLlibresIndicadors/LlibreMortalitat_2012.html.

Ainsworth BE, Haskell WL, Herrmann SD, Meckes N, Bassett DR, Tudor-Locke C, et al. 2011. 2011 Compendium of Physical Activities: a second update of codes and MET values. Med. Sci. Sports Exerc. 43:1575–81; doi:10.1249/MSS.0b013e31821ece12.

Bartoll X, Salvador M, Allué N, Borrell C. 2013. Enquesta de Salut de Barcelona 2011. Available: http://www.aspb.cat/quefem/docs/Informe_Salut_2011.pdf.

European Environment Agency. 2007. Urban Atlas.

Gascon M, Triguero-Mas M, Martínez D, Dadvand P, Rojas-Rueda D, Plasència A, et al. 2016. Residential green spaces and mortality: a systematic review. Environ. Int. 86:60–67.

Generalitat de Catalunya. 2006. Mapa estratègic de soroll del Barcelonès I.

Gobierno de España. 2012. Atlas de la Vulnerabilidad Urbana en España. Realizado en base al Censo de Población y Viviendas de 2001.

Grupo de Investigación Kraken. Universidad Extremadura. 2007. Mapas climáticos de España peninsular de temperaturas máximas y mínimas y precipitaciones a nivel mensual.

Guo Y, Gasparrini A, Armstrong B, Li S, Tawatsupa B, Tobias A, et al. 2014. Global variation in the effects of ambient temperature on mortality: a systematic evaluation. Epidemiol 25:781–9; doi:10.1097/EDE.0000000000000165.

Halonen J, Hansell A, Gulliver J, Morley D, Blangiardo M, Fecht D, et al. 2015. Road traffic noise is associated with increased cardiovascular morbidity and mortality and all-cause mortality in London. Eur. Heart J. 36:2653–2661; doi:10.1093/eurheartj/ehv216.

IPAQ Webpage. 2005. Guidelines for Data Processing and Analysis of the International Physical Activity Questionnaire (IPAQ) – Short and Long Forms.

Klein Tank A. 2002. Daily dataset of 20th-century surfaceair temperature and precipitation series for the European Climate Assessment. Int. J. Clim. 22: 1441–1453.

Krewski D, Jerrett M, Burnett R, Ma R, Hughes E, Shi Y, et al. 2009. Extended follow-up and spatial analysis of the American Cancer Society study linking particulate air pollution and mortality. Res. Rep. Heal. Eff. Inst. 140: 5–114.

Nelson ME, Rejeski WJ, Blair SN, Duncan PW, Judge JO, King AC, et al. 2007. Physical activity and public health in older adults: recommendation from the American College of Sports Medicine and the American Heart Association. Circulation 116:1094–105; doi:10.1161/CIRCULATIONAHA.107.185650.

Woodcock J, Franco OH, Orsini N, Roberts I. 2011. Non-vigorous physical activity and all-cause mortality: systematic review and meta-analysis of cohort studies. Int. J. Epidemiol. 40:121–38; doi:10.1093/ije/dyq104.

World Health Organization. 2006. WHO Air quality guidelines for particulate matter, ozone, nitrogen dioxide and sulfur dioxide. Global update 2005. Summary of risk assessment. Available: http://apps.who.int/iris/bitstream/10665/69477/1/WHO_SDE_PHE_OEH_06.02_eng.pdf.

World Health Organization. 2014. WHO Expert Meeting: Methods and tools for assessing the health risks of air pollution at local, national and international level. Available: http://www.euro.who.int/en/health-topics/environment-and-health/air-quality/publications.

World Health Organization. 2015a. Metrics: Population Attributable Fraction (PAF). Heal. Stat. Inf. Syst. Available: http://www.who.int/healthinfo/global_burden_disease/metrics_paf/en/.

World Health Organization. 2015b. The Health and Enviroment Linkages Inititative (HELI) - Quantitative assessment of environmental health impacts at population level. Available: http://www.who.int/heli/tools/quantassess/en/.
